# Synthesis and characterization of dodecylamine-capped ultrasmall metallic palladium nanoparticles (2 nm)

**DOI:** 10.1039/d5na00528k

**Published:** 2025-08-28

**Authors:** Niklas Kost, Oleg Prymak, Kateryna Loza, Christine Beuck, Peter Bayer, Claudia Weidenthaler, Marc Heggen, Cristiano L. P. Oliveira, Matthias Epple

**Affiliations:** a Inorganic Chemistry and Centre of Nanointegration Duisburg-Essen (CENIDE), University of Duisburg-Essen Universitaetsstr. 5-7 45117 Essen Germany matthias.epple@uni-due.de; b Structural and Medicinal Biochemistry, University of Duisburg-Essen Universitaetsstr. 2-5 45117 Essen Germany; c Max-Planck-Institut für Kohlenforschung 45470 Mülheim an der Ruhr Germany; d Ernst Ruska Centre for Microscopy and Spectroscopy with Electrons Forschungszentrum Jülich 52428 Jülich Germany; e Institute of Physics, University of São Paulo São Paulo 05508-090 Brazil

## Abstract

Dodecylamine-coated ultrasmall palladium nanoparticles were prepared by reduction of dichlorido(1,5-cyclooctadiene)palladium(ii) with *tert*-butylamine borane in benzene. The synthesis yielded several tens of milligrams per batch. The nature of the metal core as well as the ligand shell was elucidated by a combination of complementary methods. The particles had a diameter of about 2 nm (metallic core; by small-angle X-ray scattering and transmission electron microscopy) and a hydrodynamic diameter of about 5.4 nm (by ^1^H-NMR DOSY in benzene). They were easily dispersible in organic solvents. The ligand shell was thoroughly investigated by ^1^H and ^13^C NMR spectroscopy in dispersion, revealing about 170 ligand molecules on the surface of each 2 nm particle. X-ray powder diffraction (Rietveld refinement) and total-scattering pair distribution function analysis (PDF) showed metallic palladium nanoparticles with a crystallite size of about 2 nm, indicating a mostly single-domain nature of the nanoparticle core. X-ray photoelectron spectroscopy confirmed metallic nanoparticles also but detected oxidized palladium species.

## Abstract 2

Dodecylamine-coated ultrasmall palladium nanoparticles were prepared by reduction of dichlorido(1,5-cyclooctadiene)palladium(ii) with *tert*-butylamine borane in benzene.

## Graphical abstract

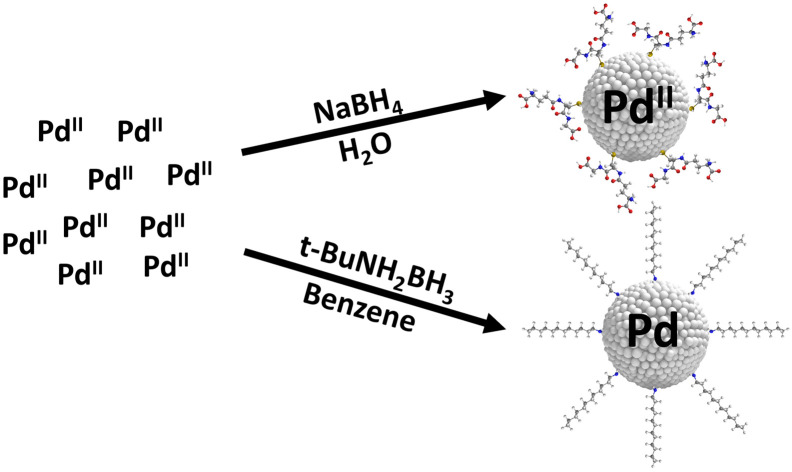

## Introduction

Nanoparticles of noble metals are of interest because they have different properties compared to the corresponding bulk phase. This is mainly due to their high specific surface area and the distinct electronic properties, which make them interesting for applications in catalysis,^1–4^ optics,^1,5^ electrochemistry,^6^ and nanomedicine.^7–12^ Palladium nanoparticles are especially interesting in heterogeneous catalysis, *e.g.* in hydrogenation or cross-coupling reactions.^3,13–15^ Ultrasmall nanoparticles are smaller than 3 nm and have an especially high specific surface area.^16–20^ Such particles consist of only several hundred metal atoms, bordering atomically sharp metal clusters with a defined structure and stoichiometry that are well known in palladium chemistry.^15,21–28^ Ultrasmall palladium nanoparticles are usually prepared by reduction in the Brust–Schiffrin method with peptides, thiols or polymers as stabilizing ligands.^3,29–36^ Clusters that are protected by the (Lewis) soft ligands such as trialkylphosphane or carbonyl are usually less stable in water.^27^ Laser ablation from palladium targets has also been reported, leading to palladium nanoparticles in the size range of 10–30 nm with mostly spherical shape and metallic character.^37^ Bönnemann *et al.* have prepared a range of different palladium nanoparticles, also by reduction with tetraalkylammonium hydrotriorganoborates in coordinating organic solvents, obtaining particle sizes of several nanometers.^38^

The colloidal stabilization of nanoparticles is a major aspect when it comes to their synthesis and application. Usually, suitable capping agents are attached to the nanoparticle surface to prevent particle growth and agglomeration. Usually, such capping ligands are attached to the particle surface *via* stable adsorption (*e.g.* polymers and polyelectrolytes) or *via* covalent bonding between the ligand and the particle surface. We can also distinguish between hydrophilic nanoparticles, which are usually prepared in water, and hydrophobic nanoparticles, which are usually prepared in organic solvents. Hydrophilic nanoparticles are often not dispersible in hydrophobic (organic) solvents and *vice versa*. The DLVO theory by Derjaguin, Landau, Verwey and Overbeek was developed in the 1940s to describe the interactions of dispersed particles.^39^ Many syntheses of colloidally stable nanoparticles have been reported, but often the yield is low and the reproducibility is poor (see ref. 38 and 40–50 for authoritative review articles on these topics with a focus on metal nanoparticles).

Palladium nanoparticles tend to oxidize in contact with air or water. Therefore, water-based syntheses of ultrasmall palladium nanoparticles can lead to ill-defined partially oxidized species as demonstrated by X-ray photoelectron spectroscopy (XPS),^29,51,52^ electron diffraction,^29^ and X-ray powder diffraction.^29^ Mao *et al.* prepared ligand-free palladium nanoparticles on carbon supports with a diameter of about 2 nm, using NaBH_4_ and amine borane as reducing agents. XPS indicated a high degree of oxidation to palladium (+II) species.^51^ Li *et al.* reduced PdCl_2_ with NaBH_4_ and deposited the ultrasmall nanoparticles (about 1.2 nm) on a carbon support. They observed strong oxidation to PdO by XPS.^52^ Gavia and Shon reviewed the application of unsupported thiol-capped palladium nanoparticles for heterogeneous catalysis but did not discuss the possible oxidation of the nanoparticles.^53^ However, palladium is often used under reducing conditions in heterogeneous catalysis, *e.g.* in hydrogenation reactions.^43,54–58^ Thus, it can be safely assumed that the initially oxidized palladium species are reduced under these catalytic conditions.

There are three different pathways that can lead to oxidized palladium nanoparticles in the case of water-based syntheses. First, oxidation may result from hydrolysis of the palladium precursors used (such as PdCl_2_) by water. Second, oxidation of initially formed palladium nanoparticles by dissolved oxygen in aqueous dispersion may occur, eventually leading to PdO.^29^ The common reducing agent NaBH_4_ necessitates extensive washing of the nanoparticles to remove residual sodium and borate ions, *i.e.* a procedure that involves extensive contact with water. Third, palladium may react with sulphur from a thiol-terminated capping ligand and form PdS. The elimination of hydrogen sulphide (H_2_S) from a glutathione capping ligand has been reported for ultrasmall silver nanoparticles during dispersion in water, leading to Ag_2_S.^59^ This may also occur with palladium, given the similar electrochemical standard potentials (+0.80 V for Ag *vs.* +0.91 V for Pd).

Oxidation and hydrolysis can be avoided if all reactions are carried out in non-aqueous solvents without thiolated ligands under strict exclusion of water and air. Here we report on the synthesis of ultrasmall palladium nanoparticles by reduction of dichlorido(1,5-cyclooctadiene)palladium(ii) as a metal–organic precursor by *tert*-butylamine borane in the presence of the capping ligand dodecylamine in the solvent benzene. This synthesis avoids all contaminations from the reducing agent and the palladium precursor and also prevents oxidation and hydrolysis due to inert gas conditions. It reproducibly yields several tens of milligrams of uniform, well-dispersed and predominantly metallic palladium nanoparticles in one batch. The application of several complementary methods sheds light on the nature of both the palladium core and the *n*-dodecylamine ligand shell.

## Results and discussion

**Fig. 1:**
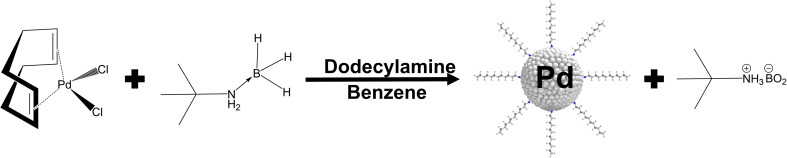
Synthesis of ultrasmall palladium nanoparticles. Dichlorido(1,5-cyclooctadiene)palladium(ii) is reduced with *tert*-butylamine borane in benzene and stabilized by the capping ligand *n*-dodecylamine.

Ultrasmall palladium nanoparticles were prepared by reducing the metal–organic palladium precursor dichlorido(1,5-cyclooctadiene)palladium(ii) with *tert*-butylamine borane. As a capping ligand, *n*-dodecylamine was used, which is attached to the palladium surface *via* the amine group. Fig. 1 shows the reaction.

**Fig. 2:**
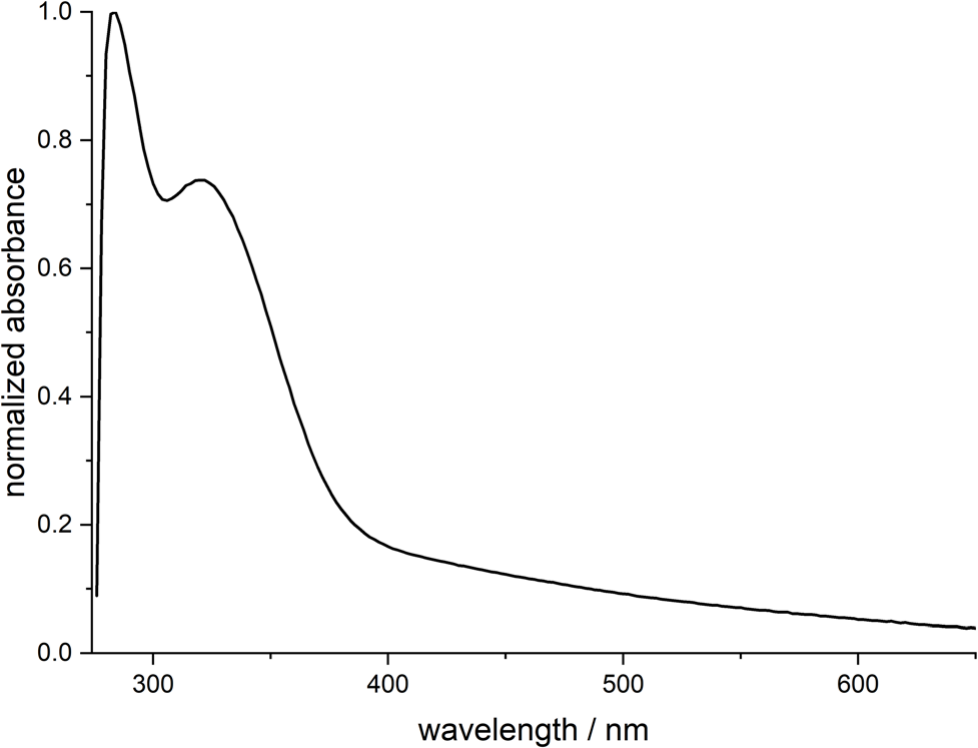
UV-vis spectra of ultrasmall dodecylamine-stabilized palladium nanoparticles, dispersed in benzene.

UV-Vis spectroscopy showed that no larger (plasmonic) nanoparticles were present (Fig. 2). A broad absorption plateau at a wavelength of around 540 nm has been described for larger palladium nanoparticles.^60^ However, this is not present for palladium nanoparticles smaller than 30 nm.^60^ The signal at approx. 300 nm results from the ligand dodecylamine (see Fig. S1), and the signal at approx. 330 nm results from the ultrasmall palladium particles.^60^

**Fig. 3:**
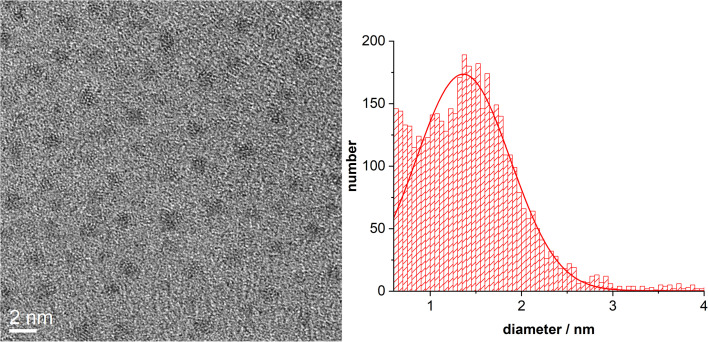
HRTEM image of ultrasmall dodecylamine-stabilized palladium nanoparticles, together with their particle size distribution (>2000 particles were analyzed).

Fig. 3 shows HRTEM images of ultrasmall palladium nanoparticles. The average particle diameter was 1.4 ± 0.5 nm with an indication for even smaller nanoparticles.

**Fig. 4:**
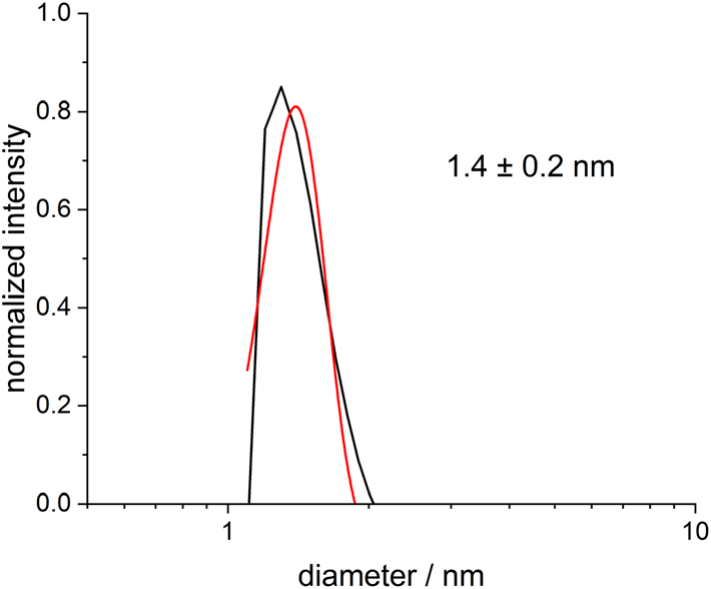
Differential centrifugal sedimentation (DCS) of ultrasmall dodecylamine-stabilized palladium nanoparticles, dispersed in benzene. DCS showed that the particles were ultrasmall and well dispersed in benzene. No aggregates or larger particles were detected. Measured data are shown in black and a Gaussian fit in red.

The determination of the particle size distribution in dispersion is possible by differential centrifugal sedimentation (DCS), which probes the hydrodynamic diameter. An average particle size of 1.4 ± 0.2 nm was found (Fig. 4), in good agreement with the HRTEM results. Note that in DCS, the particle size is systematically underestimated for ultrasmall nanoparticles, as the density of the pure metal is assumed when calculating the particle size. However, the real density of the dispersed particles is lower due to the solvated ligand shell.^61^

**Fig. 5:**
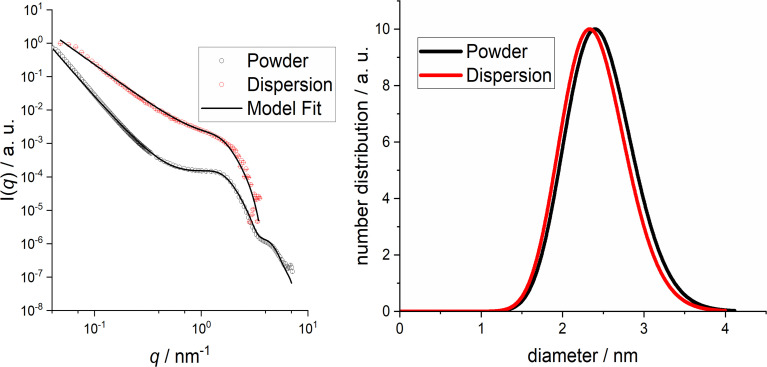
SAXS data of ultrasmall dodecylamine-stabilized palladium nanoparticles. Left: scattering data (symbols) and model fit (solid line). Right: computed particle size distributions.

Small-angle X-ray scattering is sensitive to the electron density difference between the metallic core and the dispersion. Thus, the solvated ligand shell is not probed under normal X-ray scattering conditions. Small-angle X-ray scattering data of solid (freeze-dried powder) and dispersed nanoparticles are shown in Fig. 5.

From the SAXS modelling analysis, we obtained the parameters given in Table 1. Particles with core diameters of ∼2.4 nm and a polydispersity of ∼20% were found. Furthermore, some large agglomerates were detected that can be described as fractals with overall sizes of ∼130 nm or larger. Note that the aggregate overall size is just indicative because it would be directly detectable only at even lower *q* values. For the powder samples, the formed aggregates had a fractal dimension of ∼3.0, indicating a volume fractal. Interestingly, the fractal dimension was only ∼2.2 for the dispersed samples, suggesting a surface fractal. All in all, the SAXS data confirmed the formation of spherical nanoparticles with the presence of a few larger aggregates.

**Table 1:** Model parameters of ultrasmall dodecylamine-stabilized palladium nanoparticles analysed by USAXS/SAXS

Parameters	Powder	Dispersion
*R*/nm	1.23 ± 0.03	1.2 ± 0.1
*σ*/nm	0.21 ± 0.01	0.20 ± 0.05
*D*_f_	2.99 ± 0.02	2.23 ± 0.03
*ζ*/nm	∼130	∼130
*R*_0_/nm	∼1.0	∼1.0
*R*_HS_/nm	1.65 ± 0.01	—
*η*	0.14 ± 0.02	—

NMR spectroscopy is possible for ultrasmall metal nanoparticles with organic ligands, although the NMR peaks are significantly broadened due to the vicinity of the metal core. Nevertheless, it is a powerful method to elucidate the structure and environment of the capping ligands, also because no other method is able to give such detailed structural insight.^62–64^ NMR spectroscopy on water-dispersed particles was carried out extensively with ultrasmall metal nanoparticles,^29,65–67^ but there are only a few reports on NMR spectroscopy of nanoparticles in organic solvents, *e.g.* on triphenylphosphine-stabilized gold nanoparticles (1.8 nm) dispersed in dichloromethane.^68^ Due to the hydrophobic nature of the ligand dodecylamine, the particles were dispersed and analysed in benzene-d_6_ for NMR spectroscopy.

**Fig. 6:**
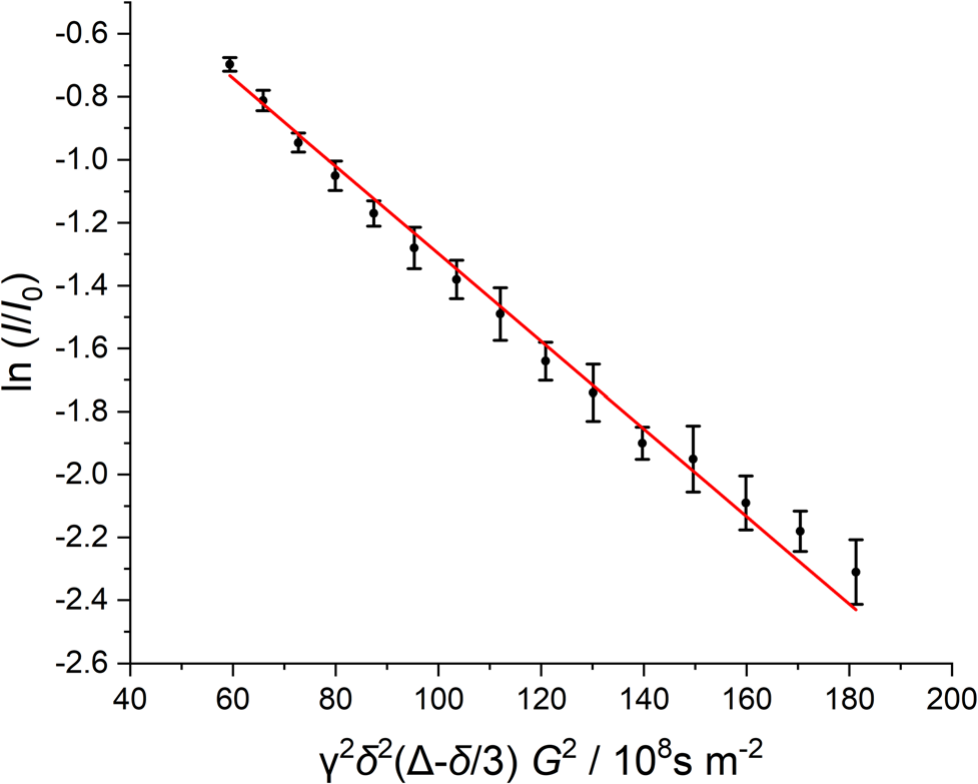
Stejskal–Tanner plot of ^1^H-DOSY NMR experiments of ultrasmall dodecylamine-stabilized palladium nanoparticles dispersed in benzene-d_6_. The data points show the average of all analysed ^1^H signals. The error bars show the standard deviation of the average. The diffusion coefficient corresponds to the negative slope.

^1^H-diffusion ordered NMR spectroscopy (^1^H-DOSY NMR) was used to determine the hydrodynamic diameter of the dispersed nanoparticles.^63,69–72^Fig. 6 shows the Stejskal–Tanner plot of the ^1^H-DOSY experiment of the benzene-dispersed nanoparticles. In general, nanoparticles diffuse more slowly than the free ligands in solution.^73^ DOSY gave a hydrodynamic diameter of 5.4 nm, highlighting the presence of the C_12_ capping ligand that extends into the dispersion. If we assume a core diameter of the metal core of 2 nm, as derived from HRTEM, SAXS, and XRD, the thickness of the solvated ligand layer is about (5.4–2) nm/2 = 1.7 nm. This demonstrates an expanded nature of the ligands on the nanoparticle surface, *i.e.* pointing into the solvent. It is in good agreement with the thickness of ligand shells of water-dispersed peptide-functionalized ultrasmall gold nanoparticles (about 0.2 nm per amino acid added to the hydrodynamic radius for linear peptides).^74^ No DOSY signals of faster diffusing dissolved ligands were found, confirming the stable attachment of dodecylamine to the nanoparticle surface.

**Fig. 7:**
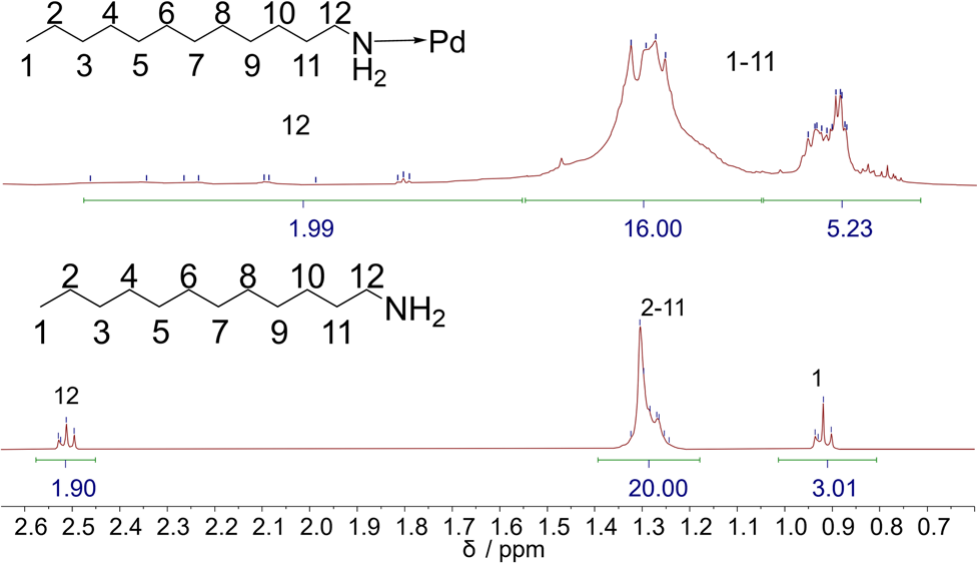
^1^H-NMR spectrum of ultrasmall dodecylamine-stabilized nanoparticles (top) and of the free ligand *n*-dodecylamine (bottom), dispersed/dissolved in benzene-d_6_, and the structure of *n*-dodecylamine with proton assignment. The peak integrals are based on the range indicated below the signals.

**Fig. 8:**
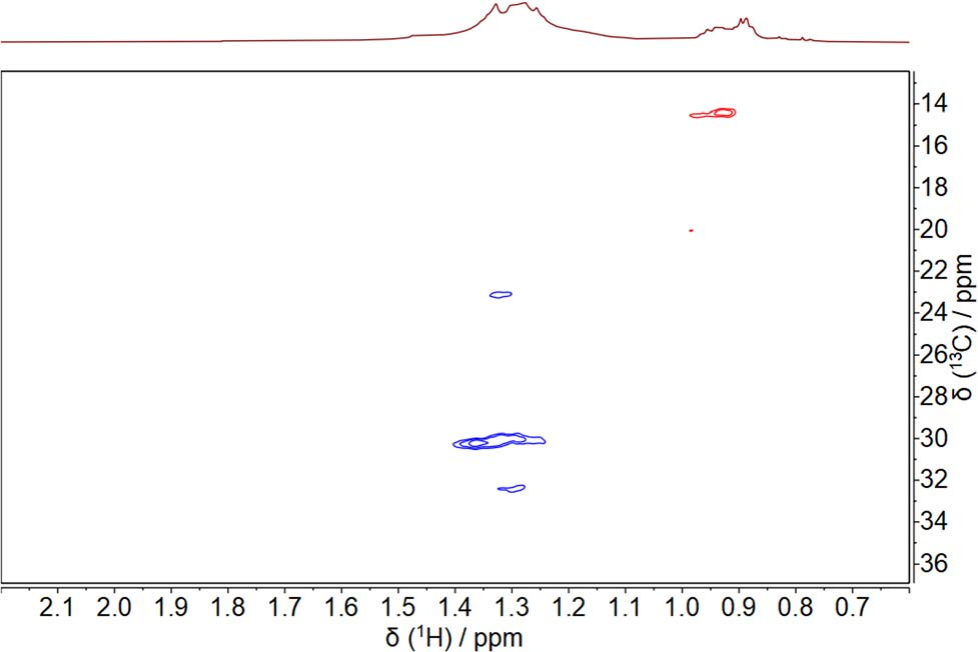
^1^H–^13^C-HSQC NMR spectrum of dodecylamine-stabilized palladium nanoparticles, dispersed in benzene-d_6_. In the HSQC spectrum, the blue color indicates CH_2_ groups, and the red color indicates the terminal CH_3_ group.

The ^1^H-NMR spectrum of the nanoparticles showed roughly the same signals as pure dodecylamine but with strongly broadened peaks (Fig. 7). The H12 methylene protons (1.7 to 2.5 ppm) were split into a number of signals, possibly due to different binding sites on the palladium nanoparticle.^75^ The broad and complex signals of the protons H2 to H11 could not be resolved. The signal of the terminal methyl group (H1) was strongly broadened but could be identified by a ^1^H–^13^C HSQC spectrum (Fig. 8).

The ^1^H–^13^C HSQC NMR spectrum showed that there are at least four different signals from methylene groups in the multiplet from 1.1 to 1.6 ppm and that there is a methyl group in the multiplet from 0.75 to 1.0 ppm. Unfortunately, the signals of the methylene group H12 were too weak to be resolved.

The quantification of ligands was possible by NMR spectroscopy. This required the ligand concentration, the nanoparticle concentration, and the nanoparticle diameter (assuming spherical nanoparticles). This gave the number of ligands per nanoparticle and the molecular footprint of each ligand molecule. The molecular footprint of ligands on ultrasmall nanoparticles is often surprisingly small, with 0.1 nm^2^ per ligand or less, indicating a high ligand density on the particle surface.^29,65,74,76^ Details of the computation can be found in ref. 76.

The concentration of ligands in the nanoparticle dispersion was determined with an external standard by ERETIC.^77^ In addition to the ligand concentration, the particle concentration in the dispersion must be determined. For this purpose, an average particle core diameter of 2 nm and a spherical shape were assumed, as derived from the HRTEM images (Fig. 3). For the palladium in the core, the density of the pure metal was used for all calculations. The palladium concentration in the sample was determined by AAS, which was then used to calculate the nanoparticle concentration. Each nanoparticle contains 286 palladium atoms, based on the density of palladium. The ratio of ligand concentration determined by NMR spectroscopy and the nanoparticle concentration by AAS resulted in 208 ligands per nanoparticle and a molecular footprint of 0.06 nm^2^. Analysing the nanoparticles with AAS (Pd) and CHN elemental analysis resulted in 137 ligands per nanoparticle and a molecular footprint of 0.09 nm^2^. Both measurement methods are of similar order of magnitude, *i.e.* the experimental results are in good agreement, giving on average about 170 ligands on each nanoparticle with a molecular footprint of 0.07 nm^2^. Of course, the number of ligands per nanoparticle depends on the average size that is assumed.

**Fig. 9:**
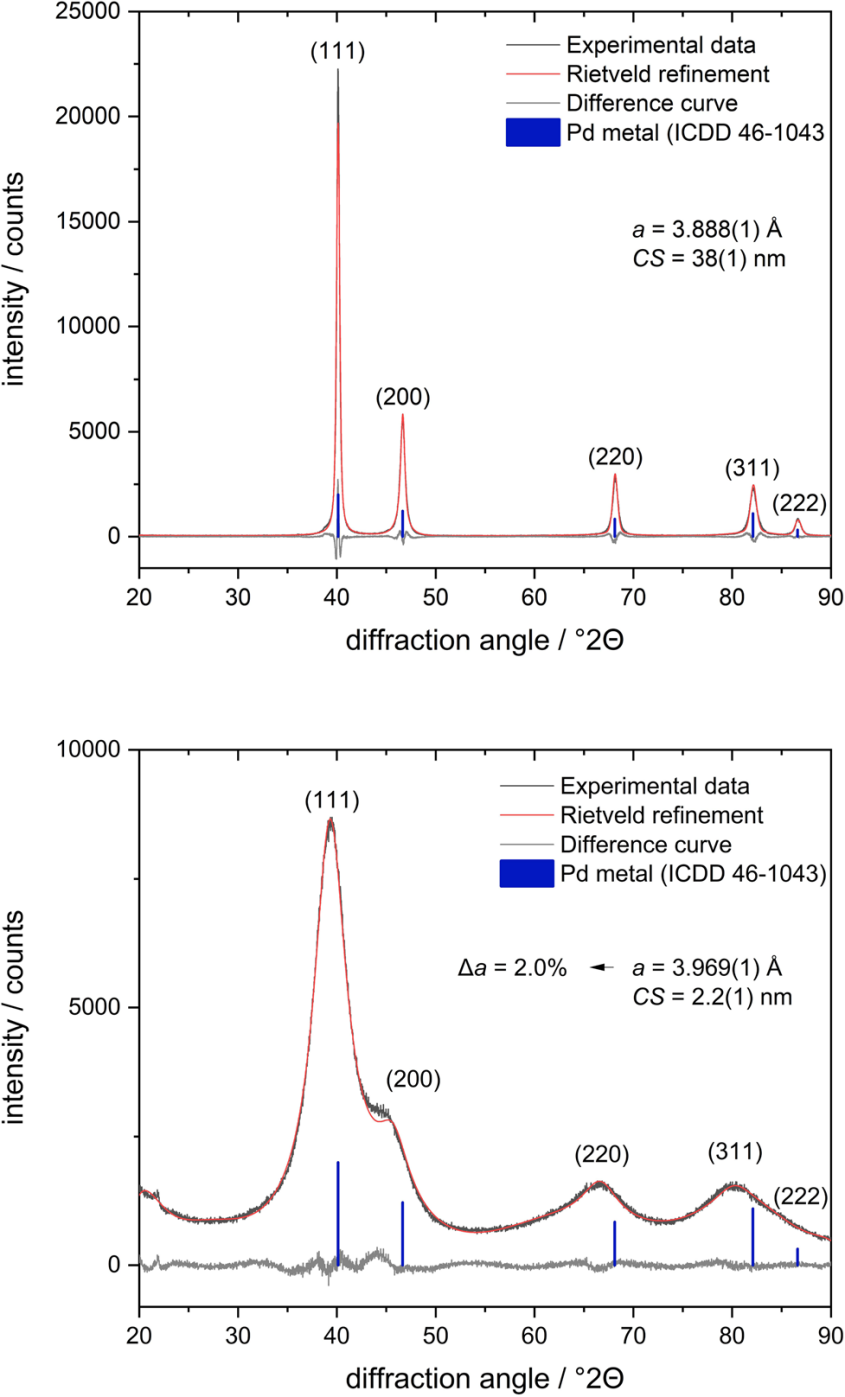
X-ray powder diffractograms of elemental palladium (top) and of ultrasmall dodecylamine-stabilized palladium nanoparticles (bottom) with Rietveld refinement (fcc; *R*_wp_ = 11.2 for palladium powder and *R*_wp_ = 3.9 for palladium nanoparticles) with the crystallite size *C*_S_. The standard deviation is given in parentheses.

X-ray powder diffraction gave insight into the crystal structure of the inorganic core of the nanoparticle (Fig. 9). The ultrasmall nanoparticles were nanocrystalline, as seen from the strong broadening of the diffraction peaks. In contrast to earlier results on water-prepared palladium nanoparticles, which showed partially oxidized palladium,^78^ the synthesis of nanoparticles in benzene with the ligand dodecylamine led to metallic palladium nanoparticles. The lattice parameter *a* was 3.969 Å, *i.e.* significantly larger than that of bulk palladium (*a* = 3.890 Å).^79^ This was confirmed by measuring a sample of elemental palladium under identical conditions, where the expected lattice constant of *a* = 3.888 Å was found (Fig. 9).

**Fig. 10:**
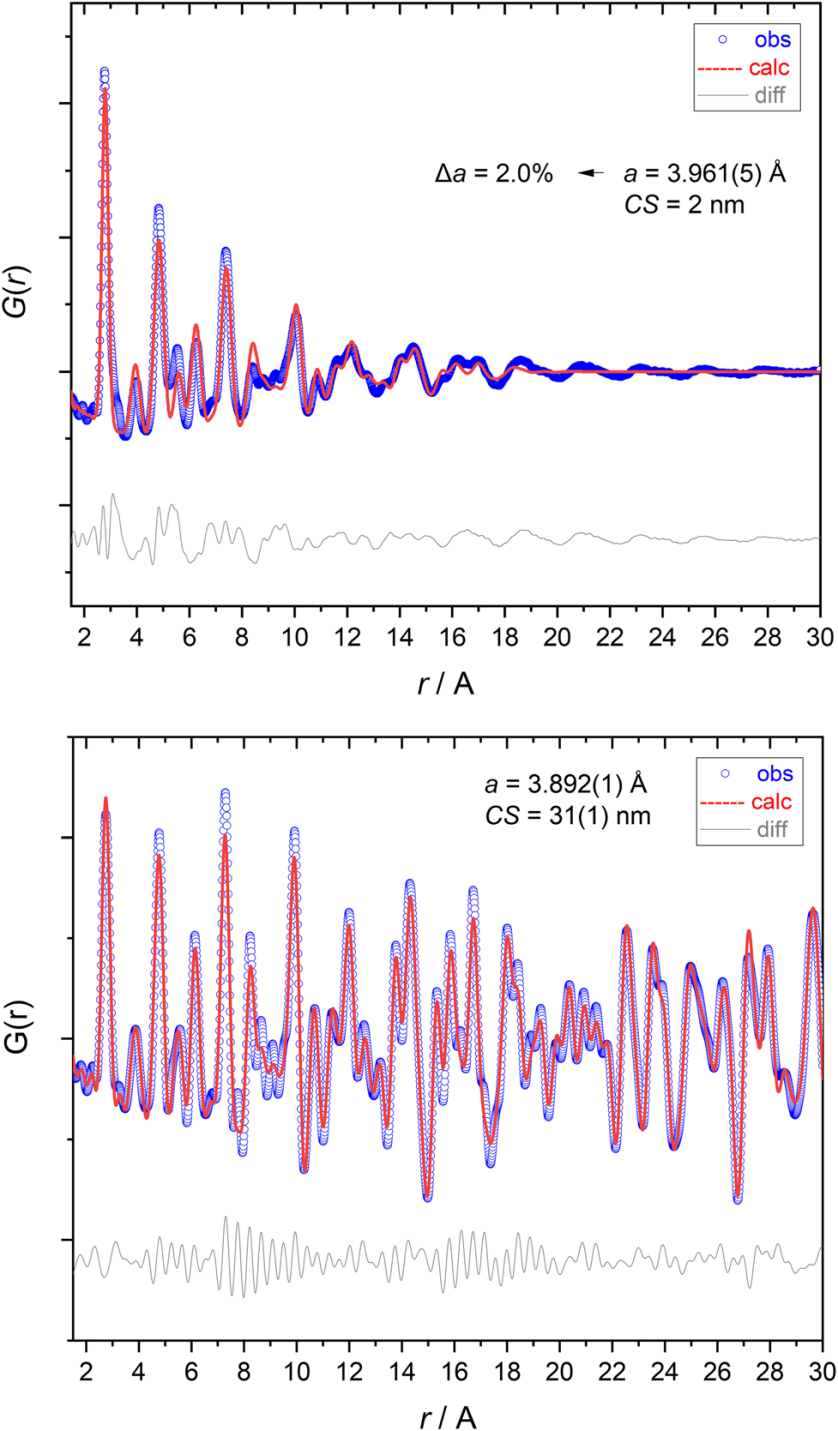
X-ray total scattering analysis (pair-distribution function; PDF) of elemental palladium (laboratory data; top) and of dodecylamine-stabilized palladium nanoparticles (synchrotron data; bottom). The standard deviation is given in parentheses.

The pair distribution function (PDF) of the dodecylamine-stabilized palladium nanoparticles showed that the particles were nanocrystalline, as the amplitude of the *G*(*r*) curve flattened out above 2 nm, indicating an estimated crystallite size of about 2 nm (Fig. 10). The crystallite size therefore corresponded well to the particle size from X-ray powder diffraction, HRTEM, and SAXS. The lattice parameter was again larger than that of the bulk material, *i.e. a* = 3.961 Å, confirming the results from Rietveld refinement. The PDF curve of elemental palladium showed the pair correlations with higher intensities to larger distances and gave the expected lattice parameter of *a* = 3.892 Å.

**Fig. 11:**
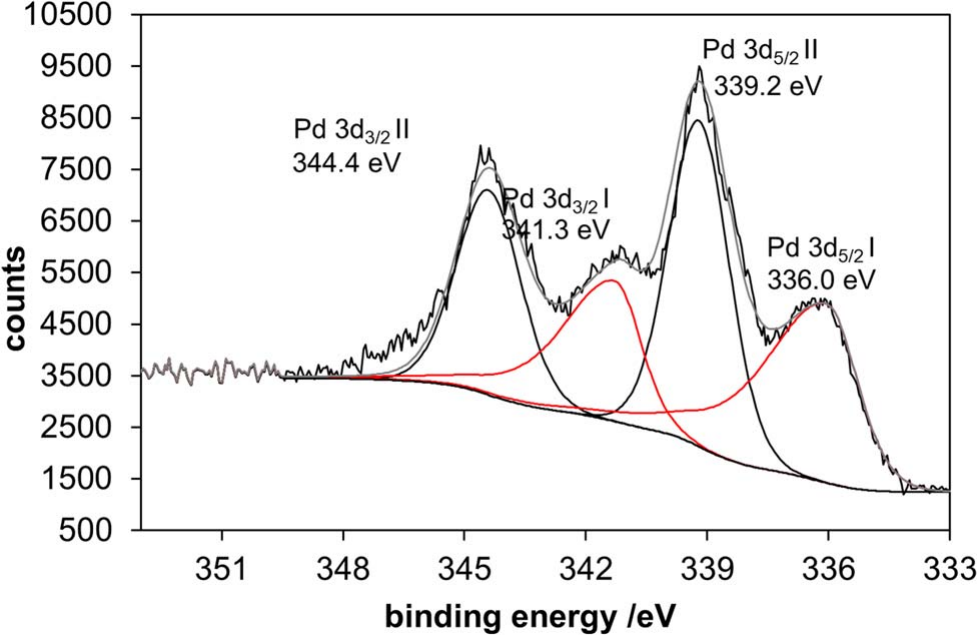
The X-ray photoelectron spectrum (XPS) of ultrasmall palladium nanoparticles shows two different palladium species: Pd-I, which is assigned to a metallic species, and Pd-II with a very high binding energy, which could be assigned to palladium oxide. Such high binding energies are usually only observed for palladium with very electronegative partners such as chloride or oxygen.

X-ray photoelectron spectroscopy (XPS) confirmed the metallic character of the palladium nanoparticles (336.0 eV), but there was also evidence for a significant degree of oxidation (339.2 eV), probably at the nanoparticle surface (Fig. 11). It could be due to reoxidation of the surface caused by handling the sample in air or by the presence of chloride, which was visible in the survey scan and may be bound to palladium. This is well in line with earlier reports on a considerable degree of surface oxidation of ultrasmall palladium nanoparticles as elucidated by XPS.^29,51,52^ The shift of the Pd 3d binding energies to higher values compared to bulk palladium can be explained by the interaction between the electronic structure of the stabilizing ligand and the metal atoms. This can influence the electron density and, consequently, the measured binding energies.^80^

Table 2 comprises all analytical data of the palladium nanoparticles.

**Table 2:** Analytical data of dodecylamine-stabilized palladium nanoparticles. The quantification of the ligand shell was performed assuming an average particle diameter of 2 nm and spherical nanoparticles

Particle parameters	Particle core volume/nm^3^	4.19
	Particle density/kg m^−3^	12 020
	Particle core weight/kg × 10^23^	5.04
	Particle core surface area/nm^2^	12.56
Particle size determination	Hydrodynamic diameter (DCS)/nm	1.4 ± 0.2
	Hydrodynamic diameter (^1^H-DOSY)/nm	5.4 ± 0.8
	Particle core diameter (SAXS)/nm	2.4 ± 0.2
	Particle core diameter (HRTEM)/nm	1.4 ± 0.5
	Crystallite size (XRD)/nm	2.2 ± 0.1
	Crystallite size (PDF)/nm	2
	Lattice parameter *a* of palladium nanoparticles (fcc; XRD)/Å	3.699 ± 0.001
	Lattice parameter *a* of palladium nanoparticles (fcc; PDF)/Å	3.691 ± 0.005
Elemental analysis	Palladium content of the nanoparticles (by AAS)/wt%	69.8
	Carbon content of the nanoparticles (by combustion analysis)/wt%	27.5
	Nitrogen content of the nanoparticles (by combustion analysis)/wt%	2.4
	Hydrogen content of the nanoparticles (by combustion analysis)/wt%	2.1
Ligand density by combination of AAS and combustion analysis	Molar ratio dodecylamine : palladium	0.48
	Dodecylamine molecules per particle	137 ± 20
	Dodecylamine molecular footprint/nm^2^	0.09 ± 0.01
Ligand density by combination of AAS and quantitative NMR spectroscopy	Molar ratio dodecylamine : palladium	0.73
	Dodecylamine molecules per particle	208 ± 30
	Dodecylamine molecular footprint/nm^2^	0.06 ± 0.01

A closer look at the particle characterization data shows that different methods give different particle size distribution results. HRTEM and SAXS both give the diameter of the metallic core, but they probe different particle populations. HRTEM can be used to analyse a comparatively low number of particles visible on an HRTEM image, whereas SAXS is an integral method that probes millions of dispersed particles in one experiment. Taking this into consideration, it is reasonable to assume a diameter of the metallic core of about 2 ± 0.5 nm based on the combined HRTEM and SAXS results, keeping in mind that SAXS also detected a small fraction of large agglomerates. In contrast, DCS and DOSY are probing the hydrodynamic diameter. While DCS systematically underestimates the particle diameter as discussed above,^61^ DOSY does not suffer from such restrictions. Therefore, the value derived from DCS primarily indicates that the particles are well-dispersed and not agglomerated.

XRD and PDF are sensitive to the internal structure of the particle core, expressed as crystallite size. They showed an excellent agreement with a crystallite size of 2.2 nm. Thus, both methods confirmed that the dodecylamine-stabilized palladium nanoparticles were metallic with an fcc structure and consisted of one crystalline domain, demonstrated by almost identical values for particle size and crystallite size. This is in contrast to ultrasmall palladium nanoparticles from water-based reduction that consisted mostly of palladium oxide (PdO).^78^ It also agrees with reports on small palladium clusters that have an icosahedral structure that turns into an fcc structure above about 90 palladium atoms, as shown in the gas phase for Pd_*n*_^−^ clusters at 95 K.^81^ The increased lattice parameter in comparison to the bulk phase is surprising but significant. Usually, nanoparticles exhibit a lattice compression, as demonstrated, *e.g.*, for ultrasmall silver nanoparticles.^29^ However, in many atom-sharp metalloid clusters, other packing types are found that are characterized by metal–metal bonds and not governed by close-packed structures.^28,44,82–87^

## Conclusions

The synthesis of ultrasmall palladium nanoparticles from a metal–organic precursor with an amine borane in benzene mostly avoids oxidation or hydrolysis to PdO and leads to predominantly metallic nanoparticles, unlike water-based syntheses. The nanoparticles have a high internal crystallinity and consist of fcc-packed palladium atoms as in the bulk metal. The lattice is expanded by about 2%. The nanoparticle size varies between 1 and 2 nm (diameter of the metallic core) and is monomodal. However, some oxidized palladium species were also detected, which is not unusual for palladium nanoparticles. The hydrodynamic diameter shows that the capping ligand dodecylamine is extending into the solvent. NMR spectroscopy shows the integrity of the capping ligand on the nanoparticle surface. The synthesis is well suited to prepare ultrasmall palladium nanoparticles for practical applications, *e.g.* in homogeneous catalysis, on a reproducible scale of several tens of milligrams per batch.

## Materials and methods

### Chemicals

We used palladium(ii)chloride (99%, Sigma-Aldrich), cyclooctadiene (99%, Thermo Scientific), palladium powder (99.95%, Thermo Scientific; grain size 0.35 to 0.8 μm), hydrochloric acid (37% in water, VWR), dodecylamine (97%, TCI), *tert*-butylamine borane (97%, Alfa Aesar), benzene (>99.5%, Carl Roth), ethanol (p.a., Fisher Scientific), diethylether (p.a., Fisher Scientific), methanol (HPLC grade, Fisher Scientific), benzene-d_6_ (99.6%, MSD Isotopes), and PolyFluor centrifugation tubes (Herolab GmbH). For differential centrifugal sedimentation (DCS), a PVC nanoparticle calibration standard (Lot#123, 263 nm, CPS Instruments Inc.) and dodecane (99%, ABCR) were used.

Ultrapure water (Purelab Ultra instrument, 18.2 MΩ, ELGA) was used if water was required. All glassware was cleaned by boiling once with concentrated nitric acid (65% in water, Fisher Scientific), followed by rinsing with water.

### Synthesis of palladium nanoparticles

A gold nanoparticle synthesis reported by Zheng *et al.*^88^ was adapted for palladium nanoparticles. Briefly, the nanoparticles were prepared with the metal–organic precursor dichlorido(1,5-cyclooctadiene)palladium(ii) as the palladium source. Dichlorido(1,5-cyclooctadiene)palladium(ii) was prepared according to King *et al.*^89^ and characterized by ^1^H- and ^13^C-NMR spectroscopy.

The nanoparticle synthesis was carried out under inert gas conditions (argon) with the Schlenk technique and dry solvents to avoid oxidation of palladium. Dichlorido(1,5-cyclooctadiene)palladium(ii) (PdC_8_H_12_Cl_2_, 57 mg, 200 μmol, 1 eq.) and dodecylamine (C_12_H_27_N, 185 mg, 1 mmol, 5 eq.) were dissolved in 100 mL benzene in a 250 mL round-bottom flask to form a clear solution. Next, a solution of *tert*-butylamine borane (C_4_H_14_BN, 199 mg, 2.3 mmol, 11.5 eq.) in 10 mL benzene was quickly added under vigorous stirring. The reaction mixture was further stirred at ambient temperature for 12 h. The palladium nanoparticles were precipitated by adding 130 mL methanol. The nanoparticles were isolated by centrifugation (4000 rpm; 2500 g; 10 min), thoroughly washed with methanol, and dried in a vacuum. The nanoparticles were easily dispersible in benzene, chloroform or dichloromethane.

### High-resolution transmission electron microscopy (HRTEM)

HRTEM was performed with an FEI TEM 80-300 microscope at an accelerating voltage of 300 kV. The nanoparticle dispersion was dripped on a copper grid coated with an ultrathin amorphous carbon film and dried in air at ambient temperature. The particle size distribution of the nanoparticles was determined with the program ANTEMA.^90^

### X-ray powder diffraction (XRD)

XRD was performed on a D8 Advance powder diffractometer (Bruker) in Bragg–Brentano reflection mode with Cu Kα radiation (*λ* = 1.5418 Å; *U* = 40 kV, *I* = 40 mA). The dried nanoparticles were placed on a single-crystal silicon sample holder to minimize scattering and dispersed with a small amount of ethanol. After drying in air, the samples were measured from 20 to 90° 2*Θ* with an increment of 0.02° and a counting time of 8 s per step, resulting in a total measurement time of 8.4 h. Qualitative phase analysis was performed with the software Diffrac.Suite EVA V7.1 (Bruker) with the pattern of Pd (#46-1043) from the ICDD database. After instrumental calibration with the reference material LaB_6_ from NIST (SRM 660b), quantitative Rietveld refinement was performed with the software TOPAS 7.0 (Bruker) to calculate the lattice parameters and the average crystallite size from diffraction peak broadening.

### Total scattering/pair distribution function (PDF)

The nanoparticles were thoroughly dried in a vacuum to remove any residual solvent. Total scattering measurements^91^ were performed at beamline ID31 at the European Synchrotron Radiation Facility (ESRF) by Momentum Transfer (Mannheim, Germany). The sample powders were loaded into cylindrical slots (approx. 1 mm thickness) held between Kapton windows in a high-throughput sample holder. Each sample was measured in transmission mode with an incident X-ray energy of 75.00 keV (*λ* = 0.1653 Å). Scattering intensities were collected with a Pilatus CdTe 2M detector (1679 × 1475 pixels, 172 × 172 μm^2^ each). The sample-to-detector distance was approximately 0.3 m. Background measurement data for the empty windows were collected and subtracted. LaB_6_ (NIST SRM 660b) was used for geometry calibration performed with the software pyFAI, followed by image integration, including flat-field, geometry, solid-angle, and polarization corrections.

Total scattering data of palladium powder were collected at room temperature on a Stoe STADI P diffractometer in transmission geometry with Mo Kα_1_ radiation (*λ* = 0.7093 Å) operated at 50 kV and 40 mA. The diffractometer was equipped with a curved Ge(111) monochromator and a Dectris Mythen 1K detector. Data were collected between 3 and 120° 2*Θ* with a step width of 0.015° 2*Θ*. The sample was prepared in a borosilicate capillary with an outer diameter of 0.5 mm. The corresponding empty capillary was also measured under the same conditions for air and background scattering correction.

The PDF data were processed with *q*_max_ = 25 Å^−1^ (synchrotron data) and 15 Å^−1^ (laboratory data). The resolution parameters were refined to values of *q*_damp_ = 0.01 Å^−1^ and *q*_broad_ = 0.015 Å^−1^. Evaluation and refinement were carried out with the programme PDFGui V1.1.^92^

### Small-angle X-ray scattering (SAXS) and ultrasmall angle X-ray scattering (USAXS)

The X-ray scattering experiments of freeze-dried powder samples were performed on a laboratory-based instrument (Xenocs-Xeuss 2.0). The X-rays were generated on a Genix3D microfocus source with Cu Kα radiation (*λ* = 1.5418 Å), focused with Fox3D mirrors and collimated with two sets of scatterless slits. The beam size was 0.7 × 0.7 mm^2^. The 2D scattering data were collected with a Dectris-Pilatus 300k detector for USAXS and SAXS, placed perpendicularly to the beam axis. The scattering images were azimuthally integrated with the program package Fit2D.^93^ A 1D curve of the scattering intensity as a function of the reciprocal space momentum transfer modulus *q*, defined as *q* = 4π sin *Θ*/*λ*, was obtained. The powder samples were sandwiched between two mica windows for the measurements. The scattering curves from the empty windows were used as blanks for the data treatment.

The same samples were measured by USAXS and SAXS. The USAXS measurements were performed at a sample-to-detector distance of 3800 mm, providing an accessible range of 0.004 < *q* < 0.105 Å^−1^. The SAXS measurements were performed at a sample-to-detector distance of 540 mm, providing an accessible range of 0.012 < *q* < 0.721 Å^−1^. SAXS and USAXS data were scaled and merged into a single curve.

Small-angle X-ray scattering experiments for the particles in dispersion were performed on an Empyrean diffractometer (Panalytical) in transmission mode with Cu Kα radiation (*λ* = 1.5418 Å, *U* = 40 kV and *I* = 40 mA, line focus) and a sample-to-detector (PIXcel3D) distance of 240 mm. A glass capillary (length 80 mm, outer diameter 1 mm, wall thickness 0.01 mm) was filled with a colloidal dispersion of palladium nanoparticles in benzene. For background correction, the same capillary was filled with benzene and measured again. The capillary was measured in a ScatterX-78 device at 1.4 × 10^−2^ mbar in the 2*Θ* range of −0.15° to 5.00° with a step size of 0.01° with a total measurement time of 21 min, providing a range of 0.01 < *q* < 0.35 Å^−1^.

The scattering data of the benzene-dispersed nanoparticles were analysed by a theoretical model that considers the nanoparticles as a polydisperse system of spheres with radius *R* and polydispersity *σ*. From the scattering curves it was derived that the particles were aggregated and, after several tests, the fractal model provided the best fit. This model comprised a fractal dimension *D*_f_, an overall fractal domain *ζ*, and a subunit radius *R*_0_. For the powder samples, strong particle–particle interactions were found, which led to repulsion structure factor effects. In this case, a hard-sphere model was used, where the interaction radius is given by *R*_HS_ and the volume fraction of the particles by *η*. This model was robust enough to describe the low-*q* dependence. The theoretical intensity used for fitting is given by

1 *I*(*q*) = *S*_C_*S*_Frac_(*q*,*D*_f_,*ζ*,*R*_0_)*S*_HS_(*q*,*R*_HS_,*η*)*P*(*q*,*R*,*σ*) + back

where *S*_C_ is an overall scale factor and back is a constant background. All mathematical details of the fitting procedure can be found in ref. 94 and 95.

### NMR spectroscopy

For NMR spectroscopy, the freeze-dried nanoparticles were redispersed in 500 μL benzene-d_6_ by ultrasonication (2 h in an ultrasonic bath). The measurements were carried out on a 600 MHz Bruker Avance III instrument at 25 °C.

### DOSY ^1^H-NMR spectroscopy

DOSY ^1^H-NMR spectroscopy on nanoparticles dispersed in benzene-d_6_ was performed on a 700 MHz Bruker NEO spectrometer with a 5 mm TCI cryoprobe with a *z*-gradient at 25 °C. Spectra were measured with a diffusion time of *Δ* = 100 ms and a pulsed gradient duration of *δ* = 3.5 ms. The gradient strength was incremented from 5 to 95% of the maximum gradient strength (64.2*G* cm^−1^ for a smoothed square gradient pulse) in 32 linear steps. The spectra were processed with the software Topspin 4.4 (Bruker). The linearized diffusion data were plotted and fitted according to the Stejskal–Tanner equation:^69,71^2
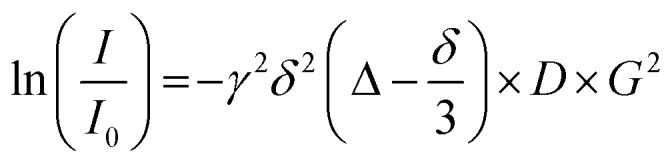
where *I* is the signal intensity, *I*_0_ is the signal intensity without gradient, *γ* is the gyromagnetic ratio of ^1^H, *δ* is the diffusion gradient pulse length, *Δ* is the diffusion delay, *G* is the gradient strength, and *D* is the translational diffusion coefficient.

The linearized relative intensities of all nanoparticle signals were averaged. The error bars represent the standard deviation of these signals. At low gradient strengths, the curve deviated from linearity due to overlapping signals resulting from small amounts of impurities with a significantly lower molecular weight. Thus, these data points were omitted from the fit. At high gradient strengths, the impurity signals had completely decayed, leaving only the intensity from the nanoparticle signals. While the standard error of the Stejskal–Tanner fit itself is small (<2%), we estimate the error in the diffusion coefficient to be 20% due to manual integration and potentially overlaying small signals from impurities.

The hydrodynamic diameter was calculated according to the Stokes–Einstein equation:^96^3
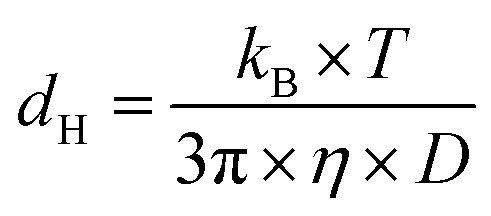
where *d*_H_ is the hydrodynamic diameter, *k*_B_ is the Boltzmann constant, *T* is the temperature in K, *η* is the dynamic viscosity at 25 °C, and *D* is the translational diffusion coefficient.

### X-ray photoelectron spectroscopy (XPS)

X-ray photoelectron spectroscopy was performed with a spectrometer from SPECS GmbH equipped with a Phoibos 150 1D-DLD hemispherical energy analyser. The monochromatized Al Kα X-ray source (*E* = 1486.6 eV) was operated at 15 kV and 200 W. For high-resolution scans, the pass energy was set to 20 eV. The medium area mode was used as the lens mode. The base pressure in the analysis chamber was 5 × 10^−10^ mbar during the experiment. To account for charging effects, all spectra were referred to C 1s at 284.8 eV.

### Elemental analysis

To determine the palladium content, the sample was dissolved in concentrated nitric acid and then diluted with water to determine the concentration by atomic absorption spectroscopy (AAS) with a Thermo Electron Corporation M-Series instrument.

For CHNS elemental analysis, the dried nanoparticles were put into tin sample holders. Vanadium pentoxide was added as a combustion additive. An EA3100 elemental analyser (Euro Vector EURO EA Elemental Analyzer) was used.

### Differential centrifugal sedimentation (DCS)

Differential centrifugal sedimentation was performed with a CPS Instruments DC 24000 disk centrifuge (24 000 rpm, 29 000 relative centrifugal force; rcf). Mixtures of two different sucrose solutions (8 and 24 wt%) were applied to obtain a density gradient. Dodecane (0.5 mL) was added as a top layer to prevent evaporation. A dispersion of poly(vinyl chloride) (PVC) with a defined hydrodynamic diameter of 263 nm dispersed in water was used for calibration before each measurement. 100 μL of the nanoparticles dispersed in benzene was injected. The density of elemental palladium (12 020 kg m^−3^) was used to calculate the hydrodynamic diameter of the nanoparticles.

### UV-vis spectroscopy

UV-Vis spectroscopy was performed with quartz glass cuvettes from 200 to 800 nm (600 μL volume) with a Genesis 50 instrument (ThermoScientific). The background measurement was performed with benzene.

## Conflicts of interest

There are no conflicts to declare.

## Supplementary Material

NA-007-D5NA00528K-s001

## Data Availability

The data supporting this article have been included as part of the SI. All other primary data are shown in the manuscript. Supplementary information: UV-Vis spectrum of *n*-dodecylamine dissolved in benzene. See DOI: https://doi.org/10.1039/d5na00528k.
