# Supplementary material for: Synthesis and characterization of dodecylamine-capped ultrasmall metallic palladium nanoparticles (2 nm)
Source: Nanoscale Adv. 2025 Aug 28;7(23):7547–58. doi: 10.1039/d5na00528k (PMC12445368; doi:10.1039/d5na00528k)
Supplement: NA-007-D5NA00528K-s001 [file NA-007-D5NA00528K-s001.pdf]

Supplementary Information (SI) for Nanoscale Advances.
This journal is © The Royal Society of Chemistry 2025

Supporting Information

Synthesis and characterization of dodecylamine-capped ultrasmall metallic palladium nanoparticles (2 nm)

Niklas Kost,[1] Oleg Prymak,[1] Kateryna Loza,[1] Christine Beuck,[2] Peter Bayer,[2] Claudia Weidenthaler,[3] Marc Heggen,[4] Cristiano L. P. Oliveira,[5] Matthias Epple[1,*]

[1] Inorganic Chemistry and Centre of Nanointegration Duisburg-Essen (CENIDE), University of Duisburg-Essen, Universitaetsstr. 5-7, 45117 Essen, Germany
[2] Structural and Medicinal Biochemistry, University of Duisburg-Essen, Universitaetsstr. 2-5, 45117 Essen, Germany
[3] Max-Planck-Institut für Kohlenforschung, 45470 Mülheim an der Ruhr, Germany
[4] Ernst Ruska Centre for Microscopy and Spectroscopy with Electrons, Forschungszentrum Jülich, 52428 Jülich, Germany
[5] Institute of Physics, University of São Paulo, São Paulo 05508-090, Brazil

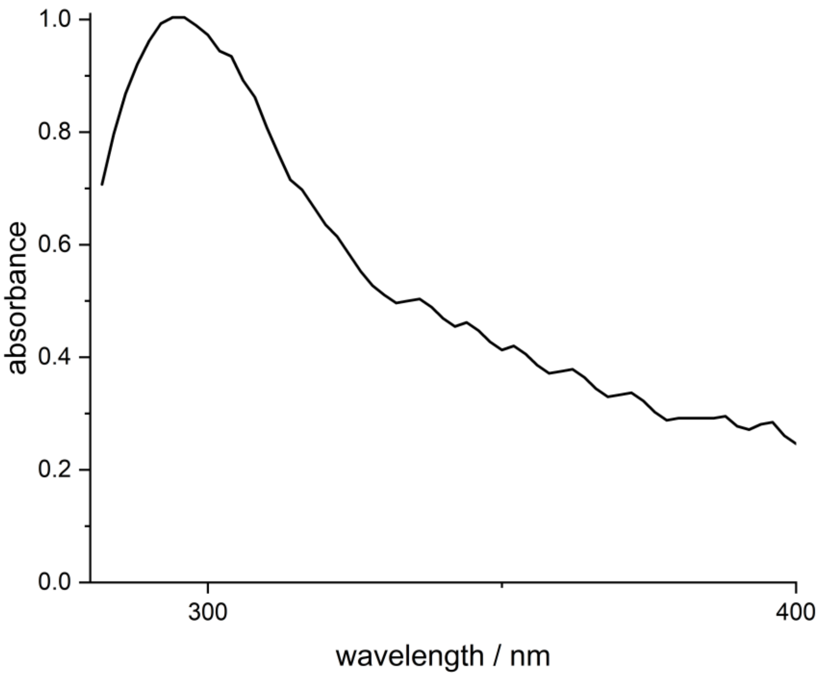

Figure S1: UV-Vis spectrum of dodecylamine, dissolved in benzene.
